# When resolution does matter: Modelling indirect contacts in dairy farms at different levels of detail

**DOI:** 10.1371/journal.pone.0223652

**Published:** 2019-10-17

**Authors:** Alba Bernini, Luca Bolzoni, Renato Casagrandi

**Affiliations:** 1 Dipartimento di Elettronica, Informazione e Bioingegneria, Politecnico di Milano, Milan, Italy; 2 Risk Analysis and Genomic Epidemiology Unit, Istituto Zooprofilattico Sperimentale della Lombardia e dell’Emilia Romagna, Parma, Italy; Atlantic Veterinary College, CANADA

## Abstract

Animal exchanges are considered the major pathway for between-farm transmission of many livestock infectious diseases. Yet, vehicles and operators visiting several farms during routine activities can also contribute to disease spread. Indeed, if contaminated, they can act as mechanical vectors of fomites, generating indirect contacts between visited farms. While data on animal exchanges is often available in national databases, information about the daily itineraries of trucks and operators is rare because difficult to obtain. Thus, some unavoidable approximations have been frequently introduced in the description of indirect contacts in epidemic models. Here, we showed that the level of detail in such description can significantly affect the predictions on disease dynamics. Our analyses focused on the potential spread of a disease in a dairy farm system subject of a comprehensive data collection campaign on calf transportations. We developed two temporal multilayer networks to model between-farm contacts generated by either animal exchanges (direct contacts) and connections operated by trucks moving calves (indirect contacts). The complete model used the full knowledge of the daily trucks’ itineraries, while the partial informed one used only a subset of such available information. To account for various conditions of pathogen survival ability and effectiveness of cleaning operations, we performed a sensitivity analysis on trucks’ contamination period. An accurate description of indirect contacts was crucial both to correctly predict the final size of epidemics and to identify the seed farms responsible for generating the most severe outbreaks. The importance of detailed information emerged even more clearly in the case of short contamination periods. Our conclusions could be extended to between-farm contacts generated by other vehicles and operators. Overcoming these information gaps would be decisive for a deeper understanding of epidemic spread in livestock and to develop effective control plans.

## Introduction

The spread of infectious diseases in livestock can cause serious negative impacts both from economic and social points of view, due to animals culling, reduced production, costs of the implemented control measures, and loss of consumer trust in the food supply chain [1–3]. The 2001 UK Foot-and-Mouth Disease (FMD) outbreak highlighted the widespread consequences of livestock epidemics, with overall costs for the public sector estimated over £2.8 billion, and around 6.5 million animals slaughtered to eradicate the infection [4]. In the context of disease management and control, quantitative epidemiological studies can play a significant role in (*a*) identifying the key factors underlining the disease transmission patterns, (*b*) supporting the design of effective biosecurity measures, and surveillance and management activities, and (*c*) predicting the effects of disease control interventions [5]. In particular, network models are commonly adopted tools to represent the spread of infectious diseases in farm systems, where nodes and links indicate, respectively, farms and the potential transmission routes of the pathogen [6,7]. Obviously, to correctly evaluate the effectiveness of preventive and control measures, a detailed knowledge of the potential pathogen transmission routes is needed.

Movement of infected animals is considered the major pathway for disease introduction and spread between livestock farms for a large number of infections [8,9]. In particular, in the early phase of the 2001 FMD outbreak, the exchange of infected animals (mainly sheep) before the imposition of national movement controls was claimed to be directly responsible of the introduction of infection into several disease-free geographical areas [10–12]. For this reason, as prescribed by EU regulations and directives (European Parliament and European Council Regulation 1760/2000/EC; Council Regulation 21/2004/EC; Council Directive 2008/71/EC), extensive databases were developed in different European countries to track the movements of farmed animals and the patterns of such movements have been largely investigated [13–17].

Diseases can also spread because of the indirect contacts: vehicles and operators visiting several farms during their routine activities can get contaminated and act as mechanical vectors between the farms they visit. Although indirect contacts are known to be less infectious than direct ones [9,18], they occur more frequently [19–23] and, thus, they can potentially play an important role in case of epidemics. Moreover, Rossi and colleagues [23] showed that the effect of direct and indirect contacts can occur on different spatial scale: while indirect contacts were responsible of the infection spread at local scale, the role of direct ones was clear only at wider spatial scale. This result confirmed what already highlighted by Brennan and colleagues [22], who interviewed 56 farmers in North-West England and found out that contiguous farms were more likely to establish social relationships, facilitating sharing of equipment, vehicles and personnel. Indirect contacts can emerge not only because of visitors but also in the case of animal movements. Indeed, besides the enhanced contamination risk due to the introduction of already infected animals in other farms, animal movements can contribute to disease spread through the trucks used in the transportation themselves [19,21,22,24–29]. Epidemiological evidence of the role of contaminated transportation vehicles in farm-to-farm spread of infections has been collected for many diseases in different species, such as swine, ovine, and bovine (see [28] and references therein). This route of transmission can be particularly effective when trucks collect animals at several farms before unloading all of them in a single site. In the context of dairy farms in European countries, the role of contaminated trucks as ways of disease transmission is especially important in the case of calf transportation, where, in a single shipment, calves are collected in several dairy farms, held together in a single vehicle and finally sent to beef farms. Conversely, in the case of adults, shipments usually involve animals coming from a single farm and trucks are cleaned and disinfected after each shipment. Data regarding on-farm truck visits is rarely registered in harmonized databases, and, to date, information has been obtained through surveys [19,21,22], reducing the potential for more in-depth analyses of their role in disease spread. Lack of data and the reduced infectiousness of indirect contacts compared to direct ones are the main reasons why animal movement networks, in general, do not explicitly consider the complete truck itineraries through the several visited farms and only account for direct operations from the loading sites to the unloading ones. By doing so, intermediate transit movements of trucks in farms without any animal unloading are neglected.

To date, indirect contacts due to transportation trucks have been included in a few disease spread models, most of them regarding the swine industry [27,29–32]. In these studies, the authors obtained from official databases [27,29] or from questionnaires [31,32] data on daily animal movements, including also the unique identifiers of the trucks in charge of the transports. Based on these data, the indirect contact network was modeled as either a two-mode network with two sets of nodes representing farms and tucks or rounds, or as the projection of such network in the space of farms [29,31]. Salines and colleagues [27] proposed a new modelling approach, that they called Transit Model (TM), accounting for the chronological sequence of loading/unloading operations in a single round.

Similar approaches have been used also to describe other types of indirect contacts occurring in livestock systems. For example, two-mode networks have been adopted to describe the relationship between farms and markets [33–35]. The one-mode projection on the farm space of these networks generates the so called commercial networks, where links between farms are due to the presence of common contractors among farms. However, this approach can lead to a coarse-grained description of the contact networks, since a common contractor does not *per se* imply common personnel and vehicles visiting a pair of farms. In addition, in absence of knowledge of the daily itineraries travelled by individual operators and vehicles belonging to a single contractor, the temporal sequences of between-farm contacts are lost. However, detailed representations of indirect between-farm contacts are only possible in the rare situations when information about the temporal sequence of the on-farm visits of each operator can be obtained [23,27].

Here, we analyzed how different levels of detail in the representation of indirect contacts may affect the description of the epidemic spread process and influence the identification of the farms responsible for generating the most severe epidemics. The final goal of this work was to compare different assumptions and modelling approaches for the description of indirect contacts and to show that they can lead to contrasting conclusions from the epidemiological point of view. Since epidemiological models are commonly used as decision support system to guide the design of biosecurity plans, this could result in non-trivial implications on disease control.

## Materials and methods

### Data

In dairy farm systems, calves are a large proportion of live animals transported. In Italy, usually, a single truck loads calves at several farms and can either unload them at other farms or, more frequently, at special facilities called *assembly centers*. Calves generally stay in assembly centers for less than 24 hours, then they are either sold to beef farms or transferred to slaughterhouses.

For this study, we considered all the nine assembly centers listed in the Italian National Bovine Database (*BDN*, Commission Decision 2006/132/EC) located in the Province of Parma (Northern Italy) and analyzed the complete calf transportation system towards them occurred during a 3-month-period, from September 1^st^ to November 30^th^, 2014. From the BDN, we extracted the following information about each of the 8,618 animals that entered the nine assembly centers during the study period: a unique ID for the animal, the IDs of the two farms of transport origin and destination, and the movement date.

As many EU countries, Italian law imposes livestock truck drivers to fill out a document for each animal loaded, here called *Modello 4* (*M4*, Council Directive 1992/102/EEC), with detailed information about the loading/unloading operations. In addition to the key elements present in the BDN, each M4 contains also the exact time of animal loading, the ID of the truck and the ID of the transportation company owning it. Based on this information, it is thus possible to reconstruct the daily round of each truck, i.e., the sequence of loading/unloading operations occurred in a single day. This allows gaining insights that might unveil the between-farm contact structure generated by consecutive on-farm visits by the same truck (defined as indirect contact network). Unfortunately, despite M4s being available in Italy since 1996 (Presidential Decree 1996/317), data recorded through this traceability system is to date mainly paper-based, therefore has never been used in disease spread models.

**Fig 1:**
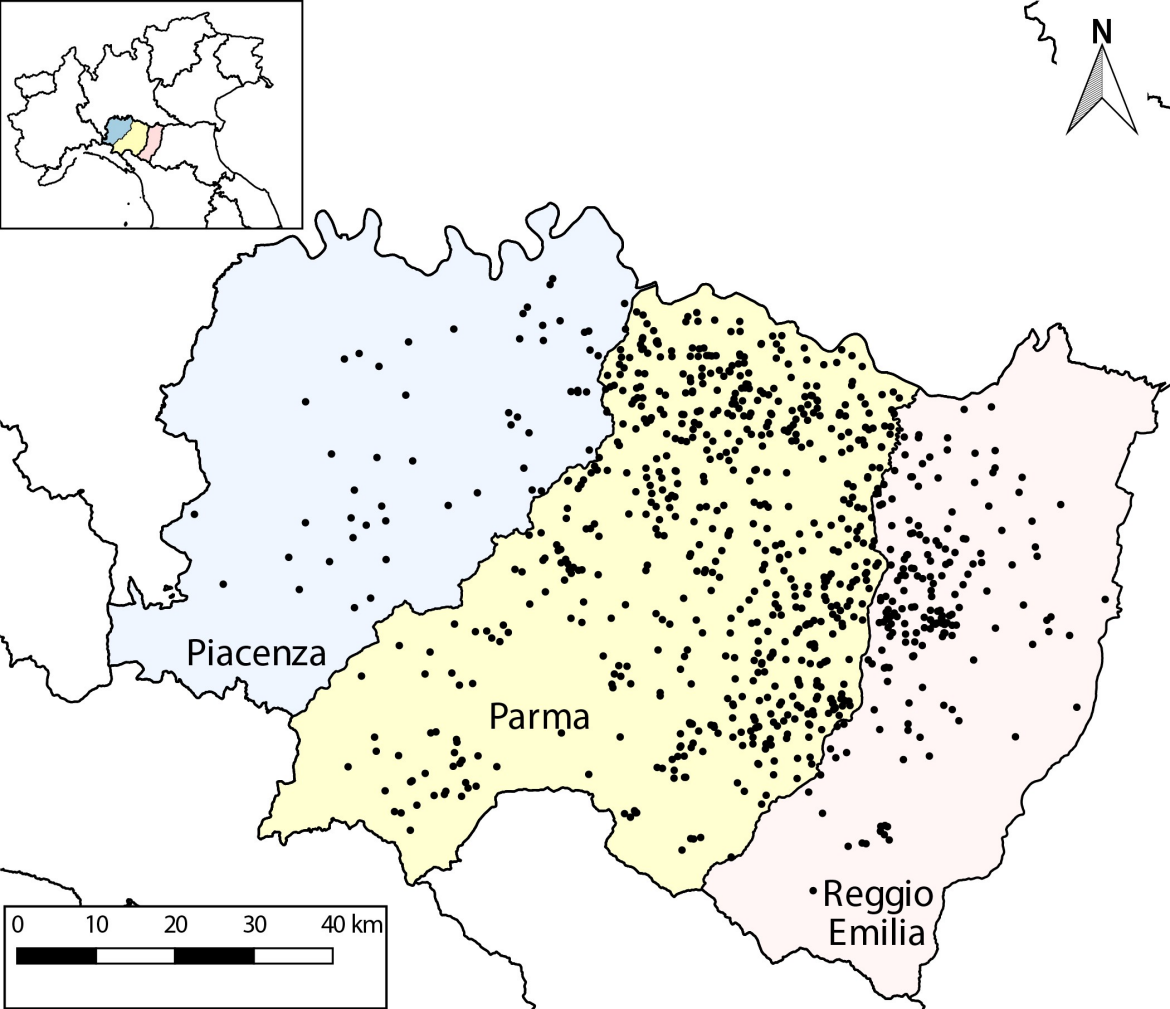
Study area and population. Dots represent dairy farms included in our analysis. A very limited set of farms (25 out of 902) is located outside the three highlighted Provinces and thus not shown. Map elaborated using data from www.istat.it.

Here, we integrated the information about calf transportation obtained from the BDN with data collected through the M4s. Only a small set of hand-filled M4s (156 out of 8,618, 2% of data) posed issues due to interpretation of incoherent information or presence of missing data and were unusable. Records describing direct movements from one assembly center to another one (914 records) were also excluded from our analysis. In such cases, groups of calves were moved without involving any farms in the system, and the health of farms is the subject of our modelling interest. In the end, the effectively usable dataset resulted made of 7,548 records of unique animal movements towards assembly centers, originating from 902 dairy farms, which constituted our study population (Fig 1).

From the BDN, we also obtained data about the farm-to-farm cattle movements (defined as direct contacts) occurred in the study population during the period of interest. In this way, we identified 736 between-farm movements, each characterized by: a unique ID of the exchanged animal, the IDs of the two farms of transport origin and destination, and the movement date.

### Network models

To describe the potentially infectious between-farm contacts generated by the movement operations of live animals, we developed two temporal multiplex network models [36,37], differing in the level of detail in the description of the indirect contacts. With 'temporal multiplex network' we intended a network that is either temporal, and thus can be interpreted as a sequence of snapshots (i.e. static networks), and multiplex, because within each snapshot links are grouped into different layers with the same set of nodes. In our study, in order to capture daily changes of the contact network structure, we built our models starting from a sequence of 91 daily snapshots (covering the period of interest). Each snapshot was composed of 902 nodes (i.e. the farms), connected by links grouped in two layers according to the type of contacts they represented (direct or indirect ones). We considered farms as discrete units, discarding any additional details about the within-farm course of infection, as commonly done in theoretical studies investigating epidemic spread in livestock systems [15,38–42].

As regards the direct contact layer, we used the dataset on cattle movement and we adopted the same modeling approach for both the network models. In each daily snapshot, a directed link was traced from a farm to another one if at least one animal was moved from the first to the second one on that day.

**Fig 2:**
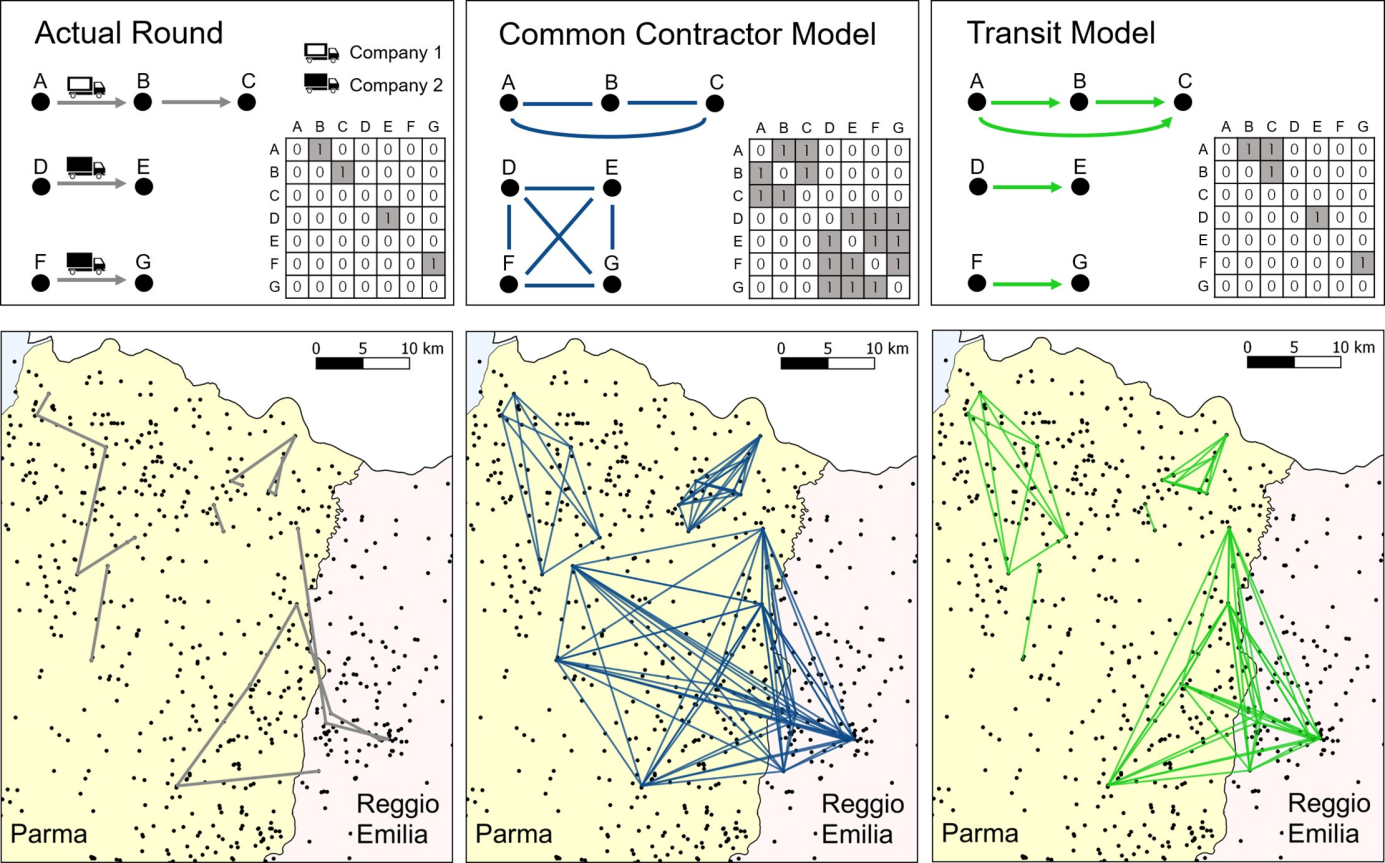
Two ways for representing indirect contacts: The Common Contractor Model (CCM) and the Transit Model (TM). The top row of panels shows a simplistic, but illustrative example, with the actual rounds of 3 trucks (left panel) belonging to 2 companies (color coded white and black, left panel). In the CCM model (central panel), all farms visited by trucks belonging to the same transportation company are connected, thus constituting a full undirected network. In the TM model (right panel), directed links are traced only from each farm to those later visited by the same truck in the same day. In this representation the contamination period is assumed equal to 0. The bottom row of panels represents the actual rounds occurred on September 9^th^, 2014 (left), together with its network representation using CCM (center) and TM (right).

The two proposed models differed in the description of indirect contacts, which were accounted for at two levels of detail, as sketched in Fig 2, but derived from the dataset on on-farm truck visits. First, we built a simpler network representation, denoted as *Common Contractor Model* (*CCM*), based only on the coarse information concerning the commercial relations between farms and transportation companies occurred at a daily temporal scale, which is commonly available for farm operations [32,43]. In this model, each daily snapshot was built projecting on the space of farms the bipartite network of commercial relations between farms and transportation companies occurred in that day (see central panels in Fig 2). Thus, farms visited in a single day by trucks belonging to a single transportation company constituted an undirected full graph. In other words, we assumed that in the CCM model the transportation company could act as mechanical vector of pathogens, regardless of how many trucks it owned. Second, taking advantage of the data derived from the M4s, we could also build a more detailed model that we called *Transit Model* (*TM*, named as such after Salines [27]). Here, a daily snapshot was built through directed links assigned from each farm to all the farms later visited by the same truck during the same day (right panels in Fig 2). The daily snapshots were built as weighted networks, both in CCM and TM. Specifically, we assigned to each link a weight equal to the number of transportation companies (in the CCM) or trucks (in the TM) responsible for that daily connection. It is worth remarking that CCM and TM differed not only in the directionality but also in the number of links, since some transportation companies owned more than one truck.

Considering as potentially infectious contacts only the links generated in the daily snapshots underlies the assumption that trucks may remain contaminated by a pathogen for a single day at most. However, the time a truck can remain contaminated, i.e. its *contamination period* (*h*), depends both on the ability of the pathogen to survive in fomites and on the frequency and effectiveness of the disinfection operations [38,44,45]. In order to assess how the contamination period *h* could affect the disease dynamics, we explored different scenarios. We assumed as a baseline the case of *h* = 0, which means that a contaminated truck could spread the infection only between farms visited in a single day. Then, we compared the simulation outcomes with what obtained with *h* = 1, 2, 3 and 4 weeks. Considering contamination periods longer than one day led to a building up of between-farm contact networks with directed links among different daily snapshots. More specifically, as regards CCM, a farm with commercial relations with a given transportation company on day *t* could be potentially infected by farms with commercial relations with the same transportation company in the previous *h* weeks. Analogously for the TM, farms visited on day *t* by a certain truck could receive the disease from farms visited by the same truck in the previous *h* weeks.

### Epidemic model

The disease spread in the farm system was modeled on both CCM and TM through a boolean Susceptible-Infectious (SI) compartmental model, where we assumed that farms could not recover once infected over the simulation time horizon. We kept the description of the disease diffusion process as simple as possible since the aim of the present study was to remark the crucial role played by the information available in building the contact networks rather than accurately describe the spread of a specific disease. Although a crude assumption, simplicity in the epidemiological (reaction) side of the process could thus help us avoiding potential confounding effects. Nonetheless, to assess the robustness of our results, in the baseline scenario (defined in the next paragraph) we simulated the disease spread on both CCM and TM with a Susceptible-Infectious-Removed (SIR) model, varying the infectious period of farms (details on the methods and on the results are reported in the S1 Text).

We first considered the baseline scenario with contamination period *h* = 0, then we repeated the simulations and the analysis on the outcomes for different values of *h* (from 1 to 4 weeks) to generalize our findings to cases of low frequency/effectiveness of disinfection operations and/or strong resistance of the pathogen in the fomites.

At the beginning of each simulation (i.e. September 1^st^, 2014), all farms were assumed to be susceptible but one, hereafter called the *epidemic seed*. Within each day, an infected farm could transmit the disease only to its susceptible nearest topological neighbors. Nodes’ status was updated only at the end of each day, which means that a node getting infected in a certain day was infectious only starting from the following day. In this way, we implicitly assumed that within a day all incoming links to a node occurred after all its outgoing ones. The transmission could independently occur through either direct or indirect contacts. Disease transmission through a direct contact was assumed to be certain, i.e., the probability of infection due to direct contacts was set equal to 1 (as in [23,42]). Conversely, we assumed a stochastic effect due to indirect contacts and we referred to probability estimates proposed by Bates et al. [18]. Specifically, for each farm *i* and each day *t*, we extracted a value *p*(*i*, *t*) of probability of infection due to indirect contacts from a BetaPERT distribution with minimum, maximum, and most likely values equal to 0.10, 0.90, and 0.50, respectively. An indirect contact from an infected farm (*I*) to a susceptible one (*J*) occurring in day *T* could succeed in transmitting the disease with probability *p*(*J*, *T*). The extraction of 91 daily values of indirect contact infection probability for each farm (corresponding to 82,082 values) was defined as *parameter setting*. For each parameter setting, we simulated the diffusion process on both CCM and TM on the complete 3-month time horizon (Sept.—Nov., 2014), seeding the disease at each farm alternatively. In other words, given a parameter setting, for each day *t* and each farm *i* we used the same probability of infection *p*(*i*, *t*) in both CCM and TM. Keeping the same epidemiological model, we could thus evaluate the effect of the network structure on the disease spread outcomes. In order to reduce the computational time, we adopted the matrix-based approach scheme proposed by Koher et al. [46], which allowed us to track the epidemics from all the possible 902 initial conditions simultaneously. To assess the epidemiological role played by each farm while acting as seed and average out the specific assignment of parameter settings, we performed 1,000 simulations from each epidemic seed, each one with a different parameter setting.

For each parameter setting and each network models, namely CCM and TM, we computed the *total epidemic size* generated by a given seed, counting infected farms at the end of the simulations, i.e. at the end of the time horizon of 91 days. We aggregated the 1,000 total epidemic sizes generated by each seed on each model, computing the median of their distribution. The farms were ranked separately in decreasing order of the median total epidemic size they generated when acting as seeds in CCM and TM, respectively. In order to assess the correlation between the two rankings, we used the non-parametric test Kendall's τ [47]. Nodes in the first 5% positions of the rankings were defined as the *most influential seeds*. We compared the CCM and TM sets of the most influential seeds, using Jaccard index [48], to evaluate how many of them belonged to the set of the most influential seeds set in both models. Finally, we investigated the emergence of recurrent invasion paths generated by the seeds on both the proposed models, extending the methodology proposed by Bajardi et al. [42] in order to account for the stochasticity of our case study. Specifically, we built the Initial Conditions Similarity Networks (ICSN), a complete weighted undirected network in which each node is an epidemic seed and the link between two nodes is weighted by a measure of the similarity of their invasion paths. We defined the *invasion path* ν^i^_tot_ of node *i* as the set of nodes that were infected at the end of at least one simulation in which node *i* acted as epidemic seed. Each node in ν^i^_tot_ was attributed a number indicating how many simulations seeded at *i* infected it. For each couple of nodes in the set of the most influential seeds, we computed the overlap between their invasion paths using the abundance-based version of the Jaccard index as described in [49]. The ICSNs were then filtered to remove links with weights smaller than a given threshold (we varied such threshold from 0.8 to 1 with increment equal to 0.005) and we evaluated the emergence of seeds’ clusters, defined as the network components.

Finally, we analyzed how the distribution of the most influential seeds in the CCM and the TM rankings were different, using the ROC curve method [50] and conventionally assuming as ground truth the classification in most influential and non-influential seeds based on the CCM ranking. To build the ROC curve, we compared the set of the CCM most influential seeds with the first *n* nodes in the TM ranking, with *n* varying from 1 to 902. For each value of *n*, we computed the True Positive Rate and the False Positive Rate respectively as the fractions of the CCM most influential seeds and of the CCM non-influential seeds included in the first *n* farms of the TM ranking (as in [51]).

## Results

### Summary of calf transportation

Overall, 558 truck rounds occurred in the study period. Each round was composed of several loading operations and at least one unloading at an assembly center. In particular, the average number of farms visited by a truck in a round was equal to 5.7, but it ranged from a minimum of 1 to a maximum of 18. The number of times a farm was visited by a calf transportation truck during the study period varied: 191 farms (21.1%) were visited only once, while only 3 farms were visited every week. On average, 2.4 calves were loaded at a single farm, while the mean total number of calves transported in a given round was 13.5. Of the total 49 trucks [36 transportation companies], on average 10.5 [7.3] and 31.2 [24.8] resulted active on, respectively, Mondays and Tuesdays, which are the two weekday in which calves were mainly collected.

### Summary of cattle movement

During the study period, 218 batches were moved between the farms in the study population. The batch dimension was on average 3.4, varying from 1 to 39. Cattle trade activities occurred almost every day and, thus, were not characterized by periodicity as the calf transportation.

### Total epidemic size and most influential seeds

**Fig 3:**
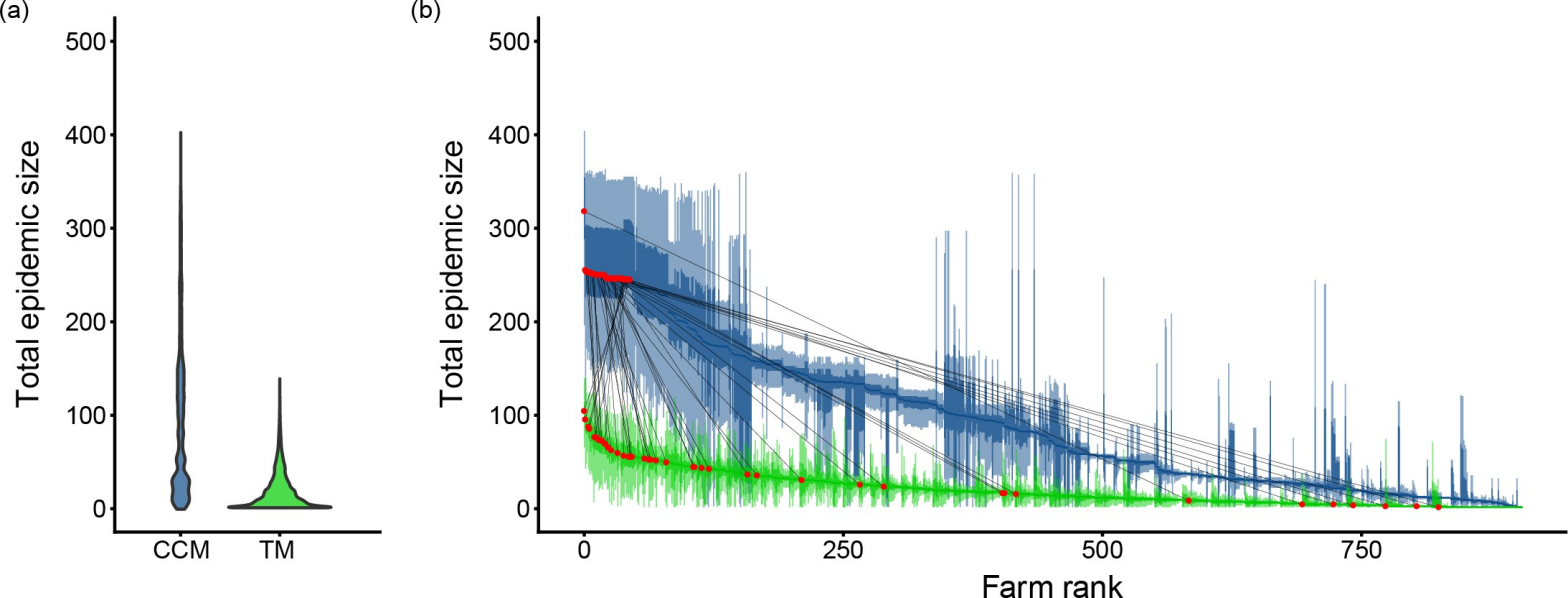
**Distribution of the total epidemic sizes obtained simulating the disease spread on the CCM (blue) and on the TM (green).** Panel A shows the violin plots (see text) describing the distributions of all total epidemic sizes obtained with the two models. In panel B, seed farms are ranked in decreasing order of the median total epidemic size they generated and each x-axis point represents a position in the rankings. The blue [green] boxplot over each x-axis point shows the distribution of the 1,000 total epidemic sizes generated by the seed in such position in the CCM [TM] ranking on the corresponding network model. Red dots represent the position of the CCM most influential seeds in both rankings. For each boxplot, the darker point is the median of the total epidemic size, the lower and upper hinges of the surrounding darker area are the first and third quartiles, respectively. The lighter area represents the whiskers: the upper [lower] whisker extends from the hinge to the largest [smallest] value no further than 1.5 x IQR from the hinge, where IQR is the interquartile range. Outliers, i.e., data beyond the end of the whiskers, are not plotted.

The results of the simulations of the SI model on our two network models in the baseline scenario of contamination period *h* = 0 are presented in Fig 3. Fig 3A shows two violin plots–i.e., mirrored density plots displayed in the same way as boxplots–representing the distributions of all 902,000 total epidemic sizes generated when the disease spread was simulated assuming that the potentially infectious contacts were drawn from CCM (in blue) and TM (in green). These two distributions were significantly different, both quantitatively and qualitatively, as confirmed by the results of the permutation test on the difference between the medians (100,000 permutations) and of the Kolmogorov-Smirnov test (p-values lower than 10^−5^ in both cases). In particular, the simulations performed using CCM led to higher values of total epidemic size with respect to the ones obtained with TM. The total epidemic sizes predicted using the CCM ranged between a minimum of 1 (when only the seed itself was infected at the end of a simulation) to a maximum of 404 infected farms (median: 64; interquartile range: 26–138). Conversely, the results obtained with the TM were comprised between 1 and 139 infected farms (median: 13; interquartile range: 4–28). Moreover, when simulations were performed using CCM, only 5 seeds resulted to be not able to infect other nodes (i.e. the total epidemic size was equal to 1 in all simulations seeded at them), while the corresponding set using TM was composed of 54 seeds. As reported in the supplementary information S1 Text, also when the disease spread was simulated using a SIR model, we obtained significantly larger total epidemic sizes in the CCM than in the TM.

The farms were ranked separately in decreasing order of the median total epidemic size they generated when acting as seeds in CCM and TM, respectively. Each point of the x-axis of Fig 3B represents a position in the ranking. Over each x point there are two boxplots: the blue one shows the distribution of the total epidemic sizes obtained in the 1,000 simulations on the CCM seeded at the farm in that position of the CCM ranking; the green one is the analogous for the TM. The rankings of the seeds derived from CCM and TM, although significantly positively correlated (Kendall’s τ = 0.41, p-value < 2.2e-16; Kendall’s τ obtained over 1,000 random comparisons: median = 0.000 and IQR = 0.031), were not at all coincident and nodes never maintained their position in both. The comparison between the sets of the most influential seeds in CCM and TM (defined as the top 5% of seeds in each ranking, i.e., positions from 1 to 45) resulted in a Jaccard coefficient equal to 0.25, corresponding to only 18 farms in common (Jaccard coefficient obtained over 1000 random comparisons: median = 0.023 and IQR = 0.023). As illustrated in Fig 3B, the set of CCM most influential seeds, which we represented by red circles in both boxplots to evidence the unexpected results obtained, were distributed all over the TM ranking. Similar findings were also obtained using an SIR model to describe the disease diffusion process, as reported in S1 Text.

**Fig 4:**
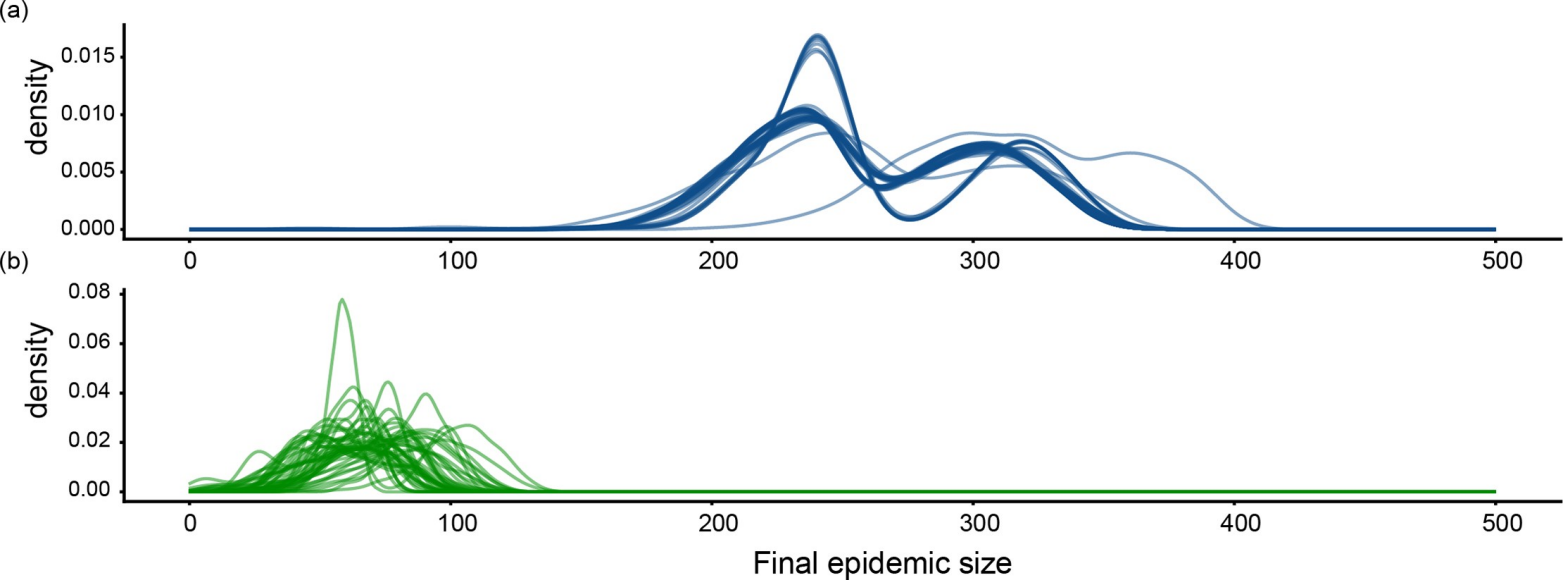
**Distribution of the total epidemic sizes generated by the CCM (panel A) and the TM (panel B) most influential seeds.** Each blue [green] curve of the top [bottom] panel shows the distribution of total epidemic sizes generated by 1,000 simulations seeded at one of the most influential seeds for the CCM [TM].

We then analyzed the characteristics of the epidemics generated by the most influential seeds identified using the CCM and the TM. Fig 4 allows a graphical comparison between the shape of the distributions of total epidemic sizes generated by 1,000 simulations seeded in the two sets of most influential seeds. The curves associated to the CCM most influential seeds (Fig 4A) appear to be grouped in clusters, while TM most influential seeds generated more heterogeneous distributions (Fig 4B).

**Fig 5:**
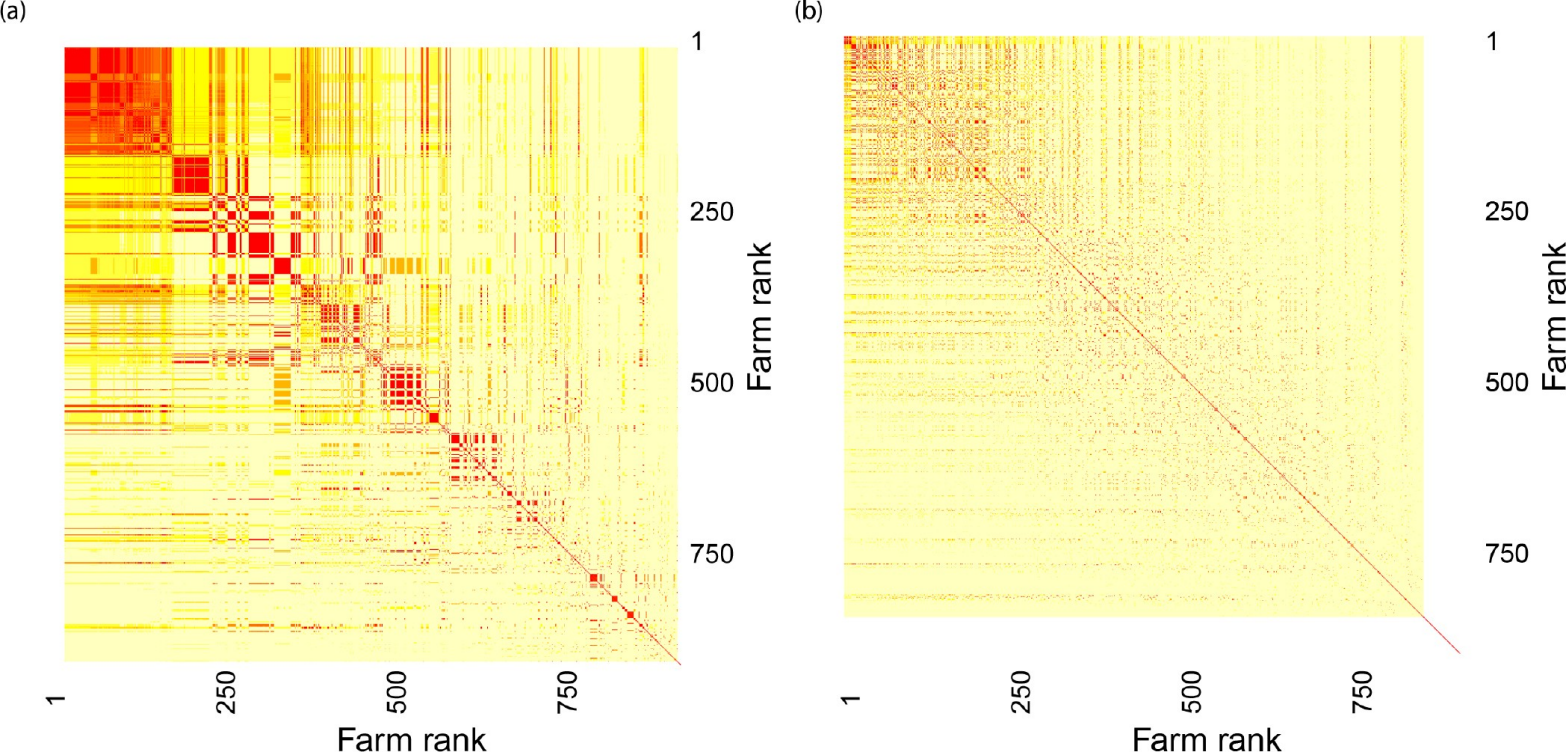
**Heatmaps showing the overlaps between the invasion paths of the seeds in the CCM (panel A) and TM (panel B) assuming a contamination period *h* = 0.** In each heatmap, the color of the position [i,j] encodes to the value of the abundance based version of the Jaccard index computed on the invasion paths generated by the seeds in the positions i and j. Nodes are sorted on the axes of the two heatmaps in decreasing order of the median total epidemic size they generated on CCM (panel A) and on TM (panel B), as done for the x-axis of Fig 3B. The last 5 rows and columns of the CCM heatmap and the last 54 rows and columns of the TM heatmap are white because the epidemics seeded at these nodes did not start (total epidemic size equal to 1) and, thus, it was not possible to compute the overlap with the invasion paths generated by the other seeds.

We further investigated the characteristics of the epidemics generated by the most influential seeds, analyzing the two Initial Conditions Similarity Networks (ICSNs). The similarities/dissimilarities between the invasion paths of all 902 seeds, i.e., the weight of the links of the ICSNs, are shown in the heatmaps in Fig 5, where seeds on the axes are ranked in the same order as on the x-axis of Fig 3B, with the most influential seeds in the top-left corner. When we filtered the two networks with a threshold value equal to 0.9, which is representative of what mostly happened within the whole range explored, the most influential seeds of the CCM and TM grouped into to 4 and the 8 different clusters respectively (the results obtained in correspondence of other thresholds are reported in the S3 Text). This results highlighted that the CCM most influential seeds generated invasion paths that were more similar than those originated by the TM ones. Thus, we could conclude that differences between the two sets of the most influential seeds emerged not only in their composition, but also in the characteristics of the epidemics they generated.

The analysis of the two heatmaps presented in Fig 5 also revealed that the invasion paths generated by the seeds when the disease spread was simulated over the CCM were in general more similar than those simulated over the TM. As regards the ICSN of the CCM (Fig 5A), red blocks can be identified along the diagonal of the heatmap, showing the presence of group of seeds characterized by high values of similarity not only within the most influential seeds but along the whole ranking. Conversely, the seeds’ invasion paths on the TM resulted to be less overlapped (Fig 5B), and, consequently, the ICSN was separated into more components. The fact that seeds’ grouped into more and smaller clusters when the disease spread was simulated over the TM showed that the epidemic outcomes were less predictable than what one would have expected according to CCM outcomes.

We repeated all the analyses also on the simulation outcomes obtained in the other scenarios considered, characterized by longer contamination periods, namely *h =* 1, 2, 3, 4 weeks.

Analogously to the baseline scenario, we used the Kolmogorov-Smirnov test to investigate the difference between the distributions of the 902,000 total epidemic sizes obtained using the CCM and the TM. The results revealed that CCM outcomes were significantly higher than TM ones in all scenarios, showing that a detailed description of the indirect contacts is important to correctly predict the potential size of an outbreak also when pathogens can survive for longer in fomites (see S2 Text). The Kolmogorov-Smirnov test was also used to perform the pairwise comparisons between the distributions of the total epidemic sizes obtained in the five scenarios with the CCM and the TM separately. As a general trend, we found that for increasing values of *h*, the total epidemic sizes predicted by both CCM and TM were significantly higher than the outcomes obtained in baseline scenario, confirming that frequent and effective cleaning and disinfection operations can limit the outbreak severity. Moreover, the CCM and the TM distributions of the total epidemic sizes obtained in the scenarios of contamination periods longer than 0 resulted more similar within them than with the ones produced in correspondence of *h* = 0 (see S2 Text).

Following the methodology adopted for the baseline scenario, we built two rankings of nodes, one for the CCM the other for the TM, for each alternative scenario. The comparison between the rankings revealed that when longer contamination periods were considered not only the sets of most influential seeds were more similar (Jaccard indices ranging in 0.64–0.80 vs the value 0.25 obtained with *h* = 0), but also the whole rankings got closer (Kendall’s τ comprised in 0.70–0.77 instead of 0.41, all p-values < 2.2e-16). The ICSN analysis revealed that at the threshold equal to 0.9, all the CCM most influential seeds were in the same cluster for the contamination periods longer than 0. A similar tendency emerged while investing the ICSN of the TM most influential seeds, where a large cluster included the majority of nodes, only few of them being apart (details in S3 Text). This means that when pathogens with longer contamination periods are accounted for, differences between the invasion paths generated by the top 5% seeds tend to become more predictable and there is a lower impact of confusing the actual rounds operated by trucks (TM approach) with usually available trajectories from commercial records (CCM).

**Fig 6:**
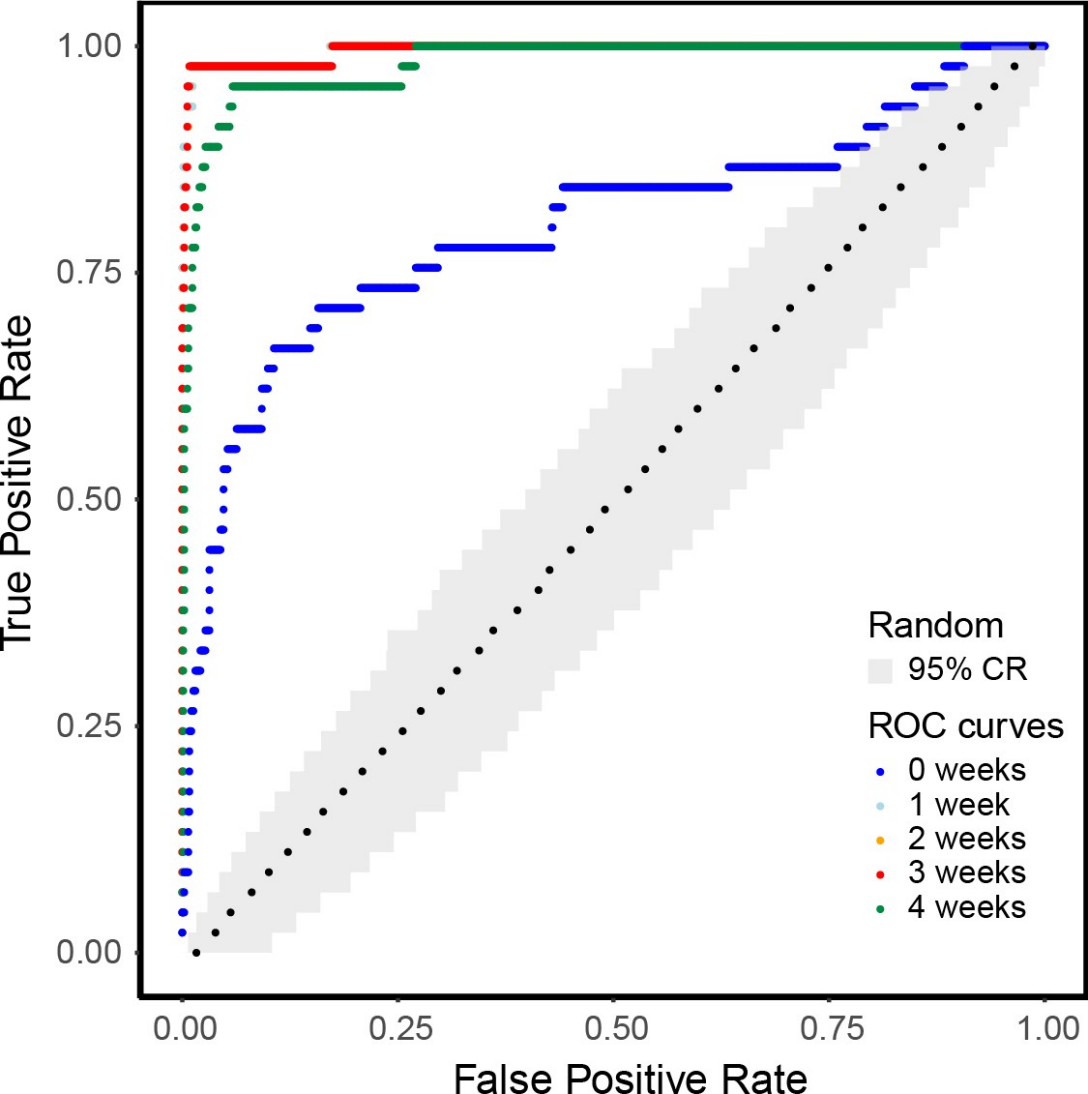
ROC curves relative to the comparison of CCM and TM classification of most influential seeds, in different scenarios of contamination period (*h*). For each value of *h* (see the color legend), we assumed as “ground truth” the classification in most influential and non-influential seeds based on the CCM ranking, i.e. based on commercial data, and we compared it with the classification based on the TM ranking (actual rounds) using the ROC curve method. The grey area represents the 95% confidence region generated on 1,000 simulations in which the TM ranking was randomly extracted. The black dots are the median behavior of a random classificatory: the x-coordinate of each point is the median of the 1,000 False Positive Rates obtained in correspondence of a value True Positive Rate.

In order to quantify to what extent the CCM and the TM rankings differed in the five scenarios of contamination period, we analyzed how the CCM most influential seeds were mapped on the TM ranking through the ROC curve (see Methods and Fig 6). If the CCM and TM classifications had been coincident for some of the analyzed contamination periods, the corresponding ROC curve would have been composed by a vertical segment, a single step in (0, 1) and then a horizontal segment to (1, 1). Therefore, the steepness of a ROC curve is closely related to similarity in between the rankings. As already pointed out while commenting the distribution of red dots in Fig 3B, in the case of contamination period equal to 0 the set of the CCM most influential seeds was well distributed over the whole TM ranking (the blue dotted line in Fig 6). Conversely, for increasing values of *h*, the ROC curves appear to be steeper, therefore there is a closer match between the more influential seeds identified by the two models.

## Discussion

The analysis of the interplay between direct and indirect contacts in the diffusion of livestock epidemics is a complex, still open issue. Many epidemiological studies considered only direct contacts [15,41,52,53], not only because they are commonly recognized as the main disease transmission route and in many cases they resulted to have the strongest influence [10,12], but also because livestock movements are fully recorded with a daily resolution in several European countries. Conversely, detailed databases about on-farm visits by personnel and vehicles are lacking. To overcome the absence of accurate information, indirect contacts have been frequently described by introducing some unavoidable approximations [33–35]. The goal of this study was to assess how and to what extent the level of detail in the description of indirect contacts could influence the outcomes of epidemic spread in farm systems, both qualitatively and quantitatively. In order to gain a general understanding of the importance of accurate information about indirect contacts, we introduced some approximations, especially in the description of the course of infection. Given these limitations, caution must be applied in the interpretation of our findings. We therefore suggest to consider the qualitative trends emerging from our analyses rather than the specific quantitative numerical outcomes.

In our study, we focused on the potentially infectious farm-to-farm contacts generated by the sharing of the trucks used in calf transportation in an important dairy farm system in the Parma province (Northern Italy). However, the outcomes we obtained might be easily generalizable to all the operators and vehicles of the livestock sector that visit several farms during their daily activities, e.g. veterinarians, hoof trimmers, deadstock collectors, AI technicians, animal feed trucks.

In livestock systems, truck sharing may play a crucial role especially in the transmission of pathogens related to highly contagious diseases, such as the foot and mouth disease, the porcine epidemic diarrhea and the Salmonella [24,32,54]. As regards the cattle industry, surveys on the routine farm activities showed that the movement of vehicles among farms could potentially play a key role in the disease diffusion, because they are associated to both not negligible probabilities of transmission and high frequency of occurrence [18,21]. However, to our knowledge, there are no studies that account for the role of trucks movements in epidemic diffusion in cattle farms with a similar level of details as investigated here. This is probably due to a substantial lack of fine scale information about the daily rounds and on-farm visits in available databases. Epidemiological models developed to describe the spread of FMD (namely, AusSpread, InterSpread Plus and the North American Animal Disease Spread Model) do incorporate in their framework different types of indirect contacts, including the ones generating by transportation trucks. However, the sequence of actual contacts occurred between farms cannot really be accounted for in those models, if not in terms of probability of links being present or not [55].

Here, we compared the outcomes obtained simulating the epidemic spread through a simple, boolean SI model on two temporal multiplex network models, differing in the description of indirect contacts generated by the calf transportation trucks. On the one hand, what we named the Common Contractor Model (CCM) was built assuming only a partial knowledge of the system (namely, the commonly available commercial relations between farms and transportation companies occurred in a day). On the other hand, our Transit Model (TM) was built by explicitly taking into account for all the rounds actually travelled by each individual truck during a period of three months. The contrasting assumptions at the basis of the two data-driven models led to differences in the number and directionality of links.

Our results showed that the modelling approach adopted for the description of indirect contacts can highly affect the simulation outcomes, both in quantitative and qualitative terms, highlighting the crucial role played by the network topology in the disease spread dynamics. The epidemic diffusion patterns predicted through the two models, namely CCM and TM, were substantially different, especially when we assumed a short time period in which trucks stay contaminated, i.e. their contamination period. On the other hand, the differences were less evident when longer contamination periods of trucks were considered, meaning that detailed information are particularly important to model all cases where pathogens are able to survive in fomites for short time period and/or in which the frequency and effectiveness of disinfection operations are high.

Numerical simulations showed that the CCM systematically led in higher values of total epidemic size, regardless of the seed farm considered (Fig 3 and S2 Text). This implies that if CCM outcomes were used to define the risk associated to large epidemics, misleading conclusions would be derived both in terms of probability of occurrence and in terms of severity. As a consequence, biosecurity measures implemented in response to this type of events would probably generate an unjustified high levels of alarmism and employed resources.

As regards the role played by seed farms in the diffusion process, we showed that the CCM and TM rankings of farms based on the total epidemic size they generated when acting as seeds were very different and that the two sets of the most influential seeds only barely overlapped. Thus, the approximated description of the epidemic spread derived from CCM failed to predict the effective role of seeds in shaping the diffusion process, which confirms the importance of a detailed reconstruction of the contact network to obtain reliable epidemiological insights. Differences were particularly evident when comparing the epidemics generated by the sets of most influential seeds in CCM and TM, in terms of both distributions of final sizes (Fig 4) and recurrence of invasion paths (Fig 5). Specifically, the outbreaks seeded at the set of CCM most influential seeds tended to display similar patterns of infected farms, while the epidemics generated by the set of TM most influential seeds showed an intrinsic variability as a function of the initial conditions of the epidemic. This result might resemble that obtained by Bajardi et al. [42] while analyzing the effect of considering different aggregating time windows in building networks of cattle exchanges. They found that using large temporal aggregations to define the contact matrices (such as monthly or yearly networks) leads to more recurring infection patterns with respect to small temporal aggregations (such as daily networks). Then, we can infer that coarse-grain representations of very different elements defining the farm-to-farm contacts, such as the temporal scale used or the farm interactions with provider companies, similarly lead to an artificial homogenization of the epidemic outcomes that does not match with the strong variability observed with fine-grain models. This finding may have significant consequences if the models were used as decision support systems for the definition of livestock disease surveillance plans. For instance, the misidentification of spreading paths that are persistent across simulations started at different initial conditions may lead to the consequent misidentification of farms having a large probability of being infected from those paths and that can logically, yet wrongly, be used as sentinels for disease surveillance. Further research should thus be conducted in this direction to assess to what extent quality and quantity of available information about livestock system in disease-free period can potentially affect the efficacy of planned biosecurity interventions in case of outbreaks.

The differences between CCM and TM became less evident when we assumed higher values of contamination period of the trucks (*h*). In these scenarios, not only the distributions of total epidemic sizes generated through CCM and TM were more similar to each other with respect to the scenario *h* = 0, but also the rankings of farms based on the total epidemic size they generated as seeds were less changeable (details in the Supporting Information). Thus, our analyses suggest that high-resolution descriptions of the between-farm contacts are particularly crucial to capture the total epidemic size in all scenarios. However, if the goal is to identify the most influential seeds less burdensome descriptions can be effectively used in cases of longer pathogen survivals.

Pathogen survival is strongly dependent on cleaning and sanitation of vehicles and equipment and these are largely considered critical biosecurity steps [44,54]. Then, according to our results, farm systems in countries with more stringent requirements on the disinfection of trucks used in the movement of live animals need better data to provide a reliable description of between-farm contacts. Our results confirmed the major impact of the assumptions on the length of fomites contamination period on simulation outcomes, as already highlighted by Rossi et al. [38] in a work focused on between-farm contacts generated by on-farm visits of veterinarians in the same geographical region considered here.

We stress that numerical simulations performed in our study were conducted using a simple boolean Susceptible-Infected model, which was not implemented with the aim of accurately describe the dynamics of specific diseases, but to help identifying general trends about modelling farm networks even in case of simple epidemiological processes. Findings were however robust against changes in the choice of the disease spread model. Indeed, the differences between the total epidemic sizes and the rankings of seeds generated with the CCM and the TM in the scenario of short contamination period were evident also when we simulated the spread process through an SIR model (details in S1 Text). Nonetheless, both the SI and SIR models we considered overlook important aspects that could be significant in the dynamics of specific epidemics. In this sense, disease-specific epidemiological compartments could be included [38,56] as well as within-farm dynamics could be taken into account in the model framework [17,56–58].

It is worth remarking that the analyses were conducted over a relatively short time horizon (three months), corresponding to the time period covered by data on trucks’ itineraries. This poses limitations on the extension of our results on long term patterns of disease spread. This limitation could be substantial in the case of marked seasonal patterns of cattle exchanges. However, the role of trucks as transmission route for infections is especially significant for the diffusion of acute and highly contagious diseases, which are commonly associated to fast spreading processes and short latent periods [24].

Our analyses were performed on a particular case study, the dairy farm system located in the Parma province (Northern Italy) and the results we obtained are valid for the particular network we considered. However, since trucks and operators working in livestock systems usually visit several farms during a single day [22,23,27,29,30], we think that our conclusions could be extended to qualitatively characterize other analogous situations.

In this study, we analyzed the role of seed farms in the process of infectious disease spread. However, it would be important to investigate also the contribution of nodes to the epidemic spread process when they are not the seeds, and this will be the topic of future analyses.

In Italy, since September 2017 data on cattle trucks’ itineraries from pilot regions began to be registered in a digitalized database at the Italian Ministry of Health (Health Minister Decree, 2016). The availability in the near future of high-quality extensive data on the on-farm visits of trucks for cattle movements at a national scale will lead to a better understanding of the role of animal exchanges in the spread of livestock diseases.

## Supporting information

S1 Text Modeling disease spread through an SIR epidemic model. (PDF)

S2 Text Distributions of the total epidemic size and of the most influential seeds in the alternative scenarios. (PDF)

S3 Text Analysis of the Initial Conditions Similarity Networks in the baseline and alternative scenarios to detect the emergence of seeds’ clusters. (PDF)

S1 Dataset Cattle movement. File related to cattle movements occurred in the study area during a 3-month-period, from September 1^st^ to November 30^th^, 2014. This dataset was derived from the information extracted from the Italian National Bovine Database (BDN). Each row represents the movement of a batch and contains the anonymized IDs of the two farms of transport origin and destination, and the movement date.
(CSV)

S2 Dataset Calf transportation. File related to on-farm visits by calf transportation trucks occurred in the study area during a 3-month-period, from September 1^st^ to November 30^th^, 2014. This dataset was obtained by integrating the information obtained from the Italian National Bovine Database (BDN) and the data collected through the documents Modello 4 (M4). Each row represents the movement of a batch from a farm to an assembly center and contains the following anonymized information: the ID of the origin farm, the ID of the destination assembly center, the movement date, the ID of the truck, the ID of the transportation company owning it and the exact time of animal loading.
(CSV)
