# Supplementary material for: When resolution does matter: Modelling indirect contacts in dairy farms at different levels of detail
Source: PLoS One. 2019 Oct 17;14(10):e0223652. doi: 10.1371/journal.pone.0223652 (PMC6797332; doi:10.1371/journal.pone.0223652)
Supplement: S1 Text — (PDF) [file pone.0223652.s001.pdf]

Supplementary information S1

When resolution does matter:
Modelling indirect contacts in dairy farms at different levels of detail

Alba Bernini, Luca Bolzoni, Renato Casagrandi

Modeling disease spread through an SIR epidemic model

In this section, we provided sensitivity analyses for the epidemic model used to simulate the disease spread. We repeated the simulations performed in the baseline scenario (contamination period equal to 0) on both CCM and TM, using a Susceptible-Infectious-Removed (SIR) epidemic model. Specifically, we assumed that disease transmission from infected to susceptible nodes occurred following the same rules of the SI model (described in the main text), but we included a recovery process. If a node became infected in a certain day t_0, after k days, i.e. the *infectious period*, it got recovered and, thus, was removed with all its future links from the network. Thus, it did not participate in the spreading process any longer. We performed also a sensitivity analysis on the infectious period, assigning it different values, namely k = 2, 3, 4 weeks.

On each network model (CCM and TM), we performed 1,000 simulations of disease spread from each seed, using the same 1,000 parameter settings considered for the SI model (as detailed in the main text). For each simulation, we computed the total epidemic size generated by the seed as the number nodes that were either infected or removed at the end of the simulation.

The boxplots in Fig S1.1 graphically illustrated that the total epidemic sizes predicted by the CCM (in blue) were significantly higher than the ones obtained with the TM (in green) in all the scenarios of infectious period. This was confirmed by the results of the permutation test on the difference between the medians (100,000 permutations) and of the Kolmogorov-Smirnov test (p-values lower than 10^{-5} in both cases in all the scenarios). Thus, the trend emerged when the disease spread process was simulated with an SI epidemic model was confirmed also when an SIR-like model was used, regardless of the infectious period.

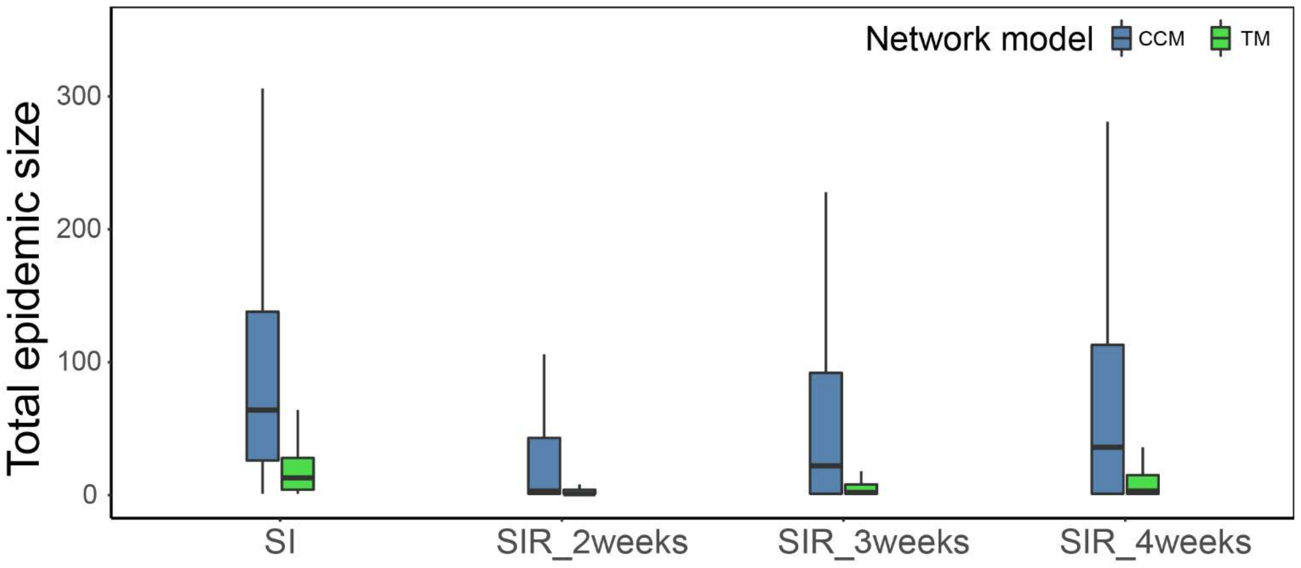

Fig S1.1. Distribution of the total epidemic sizes obtained simulating the spread of a disease on the CCM (blue) and on the TM (green) with SI and SIR models. The boxplots summarize the total epidemic sizes obtained in the baseline scenario of contamination period equal to 0, when the disease spread process was simulated using different epidemic model: an SI model (these results are analyzed in details in the main text) and an SIR model, with different assumption on the infectious period (from 2 to 4 weeks).

As regards the importance of nodes, following the same methodology adopted for the analysis of the SI simulation outcomes, for each scenario of infectious period, we generated two rankings of nodes, one for CCM, the other for TM, in decreasing order of the median total epidemic size they generated when acting as seeds. Since many seeds were not able to generate any outbreaks (probably because they recovered before their first outgoing links), we focused the attention only on the most influential seeds, i.e., the nodes on the first 45 positions of a ranking.

For each scenario, we compared the CCM and TM sets of the most influential seeds, using the Jaccard index and we obtained values ranging 0.13 – 0.25, similar to the ones obtained in the baseline scenario with the SI model. Again these findings showed that the results we obtained with the SI model were also valid for the SIR model, with different scenarios of infectious period.
