# Supplementary material for: When resolution does matter: Modelling indirect contacts in dairy farms at different levels of detail
Source: PLoS One. 2019 Oct 17;14(10):e0223652. doi: 10.1371/journal.pone.0223652 (PMC6797332; doi:10.1371/journal.pone.0223652)
Supplement: S2 Text — (PDF) [file pone.0223652.s002.pdf]

Supplementary information S2

When resolution does matter: Modelling indirect contacts in dairy farms at different levels of detail

Alba Bernini, Luca Bolzoni, Renato Casagrandi

Distributions of the total epidemic size and of the most influential seeds in the alternative scenarios

In the main text, we analyzed the results of the simulations performed assuming a contamination period of trucks (h) equal to 0. In this section, we present the distributions of the total epidemic sizes obtained in the four alternative scenarios, corresponding to contamination period lengths: 1, 2, 3, and 4 weeks. The differences between the CCM and TM distributions obtained assuming $h = 0$ (discussed in the main text) emerged also in the other scenarios. Indeed, the Kolmogorov-Smirnov test between the CCM and TM distributions confirmed that CCM total epidemic sizes were significantly higher than TM ones also for $h = 1$, 2, 3, and 4 weeks. This result was graphically supported by the distributions reported in Fig S2.1 (corresponding to Fig 3 in the main text).

Fig S2.1 obviously confirmed what already emerged from the ROC curve analysis (Fig 6 in the main text) regarding the ranking of nodes based on the median total epidemic sizes they generated when acting as seed. Indeed, the differences between the CCM and TM rankings were less evident when longer contamination periods of trucks were assumed. While in the baseline scenario ($h = 0$) the CCM most influential seeds were distributed over the whole TM ranking, in all the alternative scenarios, almost all of them were in the first 100 positions (with two exceptions in the case $h = 7$ and one in the other ones).

Then, we compared pairwisely the five distributions of total epidemic sizes generated using the CCM and the five ones obtained using the TM. Generally, it emerged that the baseline scenario ($h = 0$) led to outcomes that were strongly different with respect to the ones obtained in the other scenarios. In particular, the values of the Kolmogorov-Smirnov test statistics obtained comparing among them the CCM [TM] distributions obtained in the scenarios with longer contamination periods were lower than the values obtained comparing them to the CCM [TM] distribution obtained in the baseline scenario (see Fig S2.2).

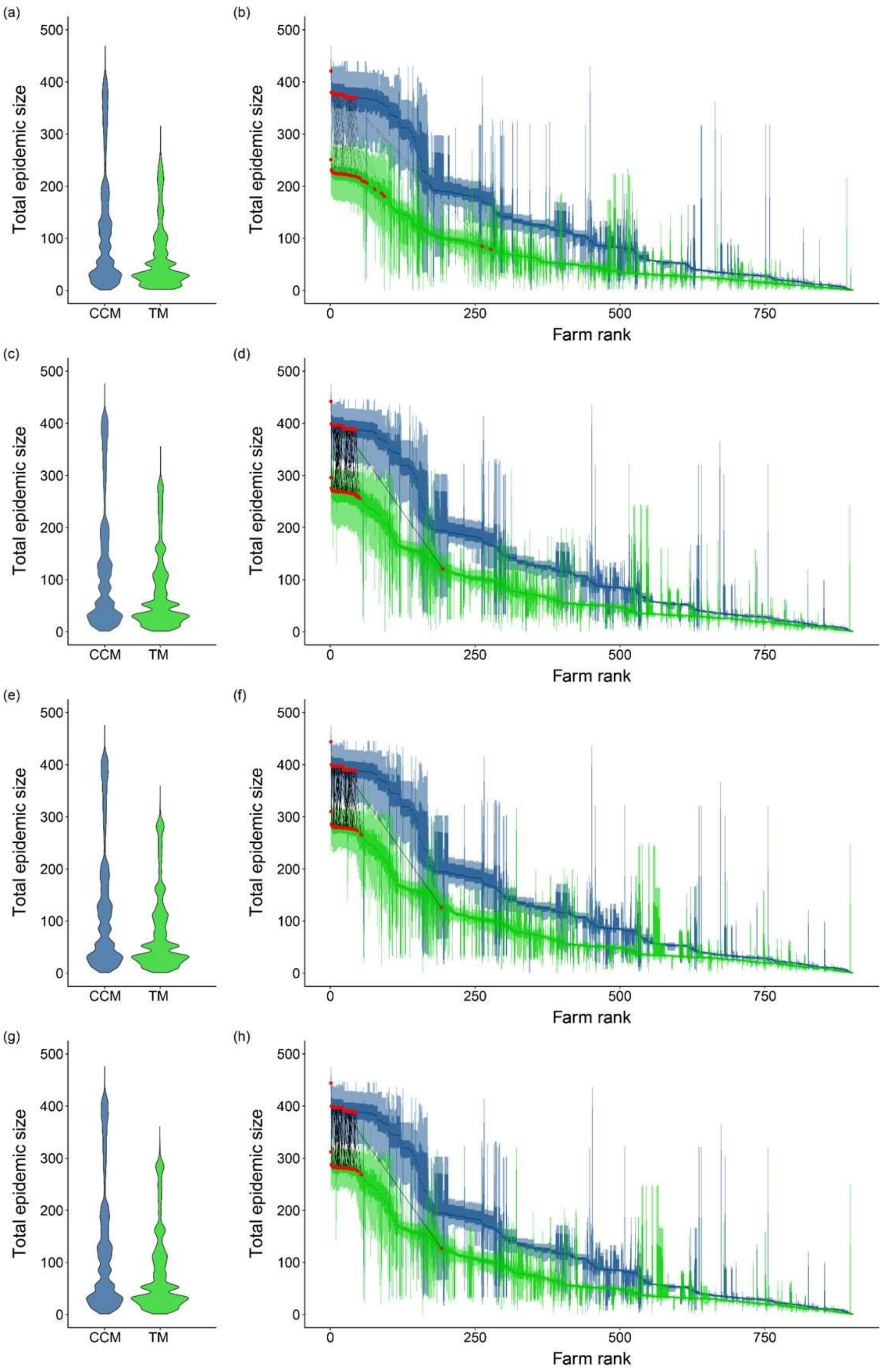

Fig S2.1. Distribution of the total epidemic sizes obtained simulating the spread of a disease on the CCM (blue) and on the TM (green) in the alternative scenarios of contamination period. The figures show the effect of the contamination period on the simulation outcomes. Panels (a) and (b): h set to 1 week; panels (c) and (d): h set to 2 weeks; panels (e) and (f) h set to 3 weeks; panels (g) and (h): h set to 4 weeks.

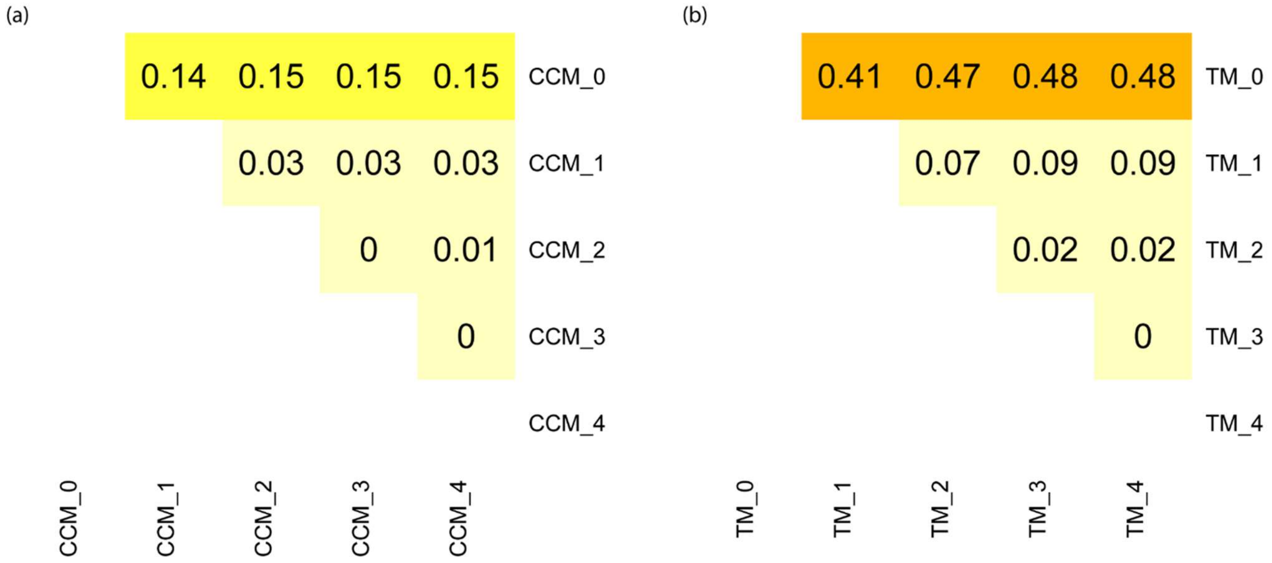

Fig S2.2. Kolmogorov-Smirnov test statistics obtained pairwisely comparing the distributions of total epidemic sizes obtained using the CCM (a) and the TM (b) in the 5 scenarios considered. In both panels, the color in the position [i,j] encodes the value of the Kolmogorov-Smirnov test statistics computed comparing the distributions of the total epidemics sizes obtained in the scenarios i and j. The p-values are less than 0.001 in all the tests, except for the comparison between the distributions obtained with the CCM in the scenarios of contamination periods equal to 3 and 4 weeks, meaning that the hypothesis that the two distributions are equal is accepted.
