# Supplementary material for: When resolution does matter: Modelling indirect contacts in dairy farms at different levels of detail
Source: PLoS One. 2019 Oct 17;14(10):e0223652. doi: 10.1371/journal.pone.0223652 (PMC6797332; doi:10.1371/journal.pone.0223652)
Supplement: S3 Text — (PDF) [file pone.0223652.s003.pdf]

Supplementary information S3

When resolution does matter: Modelling indirect contacts in dairy farms at different levels of detail

Alba Bernini, Luca Bolzoni, Renato Casagrandi

Analysis of the Initial Conditions Similarity Networks in the baseline and alternative scenarios to detect the emergence of seeds' clusters

In this section, we performed the analysis on the Initial Conditions Similarity Networks (ICSNs) in the alternative scenarios as done in the baseline one. Fig S3.1 (analogous to Fig 5 in the main text) shows the heatmaps of the comparisons between invasion paths generated by the seeds in the alternative scenarios on the two models (CCM and TM outcomes are presented in panel (a), (c), (e), (g) and (b), (d), (f), (h) respectively). As for the baseline scenario, we measured the overlap between two invasion paths using the abundance based Jaccard index. The order of the nodes on the axes of each heatmap is the same as on the x-axis of the corresponding plot in Fig S2.1.

In general, in the CCM heatmaps it was possible to identify the presence of a large group of nodes located in the first positions of the ranking that were characterized by recurrent invasion paths. The CCM heatmaps showed also the presence of groups of seeds characterized by high values of similarity along the whole ranking, analogously to in the scenario of $h = 0$ (discussed in the main text). As regards the TM, the heatmaps obtained in the alternative scenarios were different from the one obtained when $h = 0$. Indeed, recurrent invasion paths emerged and blocks along the diagonal became recognizable, although less defined that the ones in the corresponding CCM heatmaps. Moreover, in each alternative scenario, nodes in the first positions of the TM ranking appeared to belong to a unique group, with some exceptions.

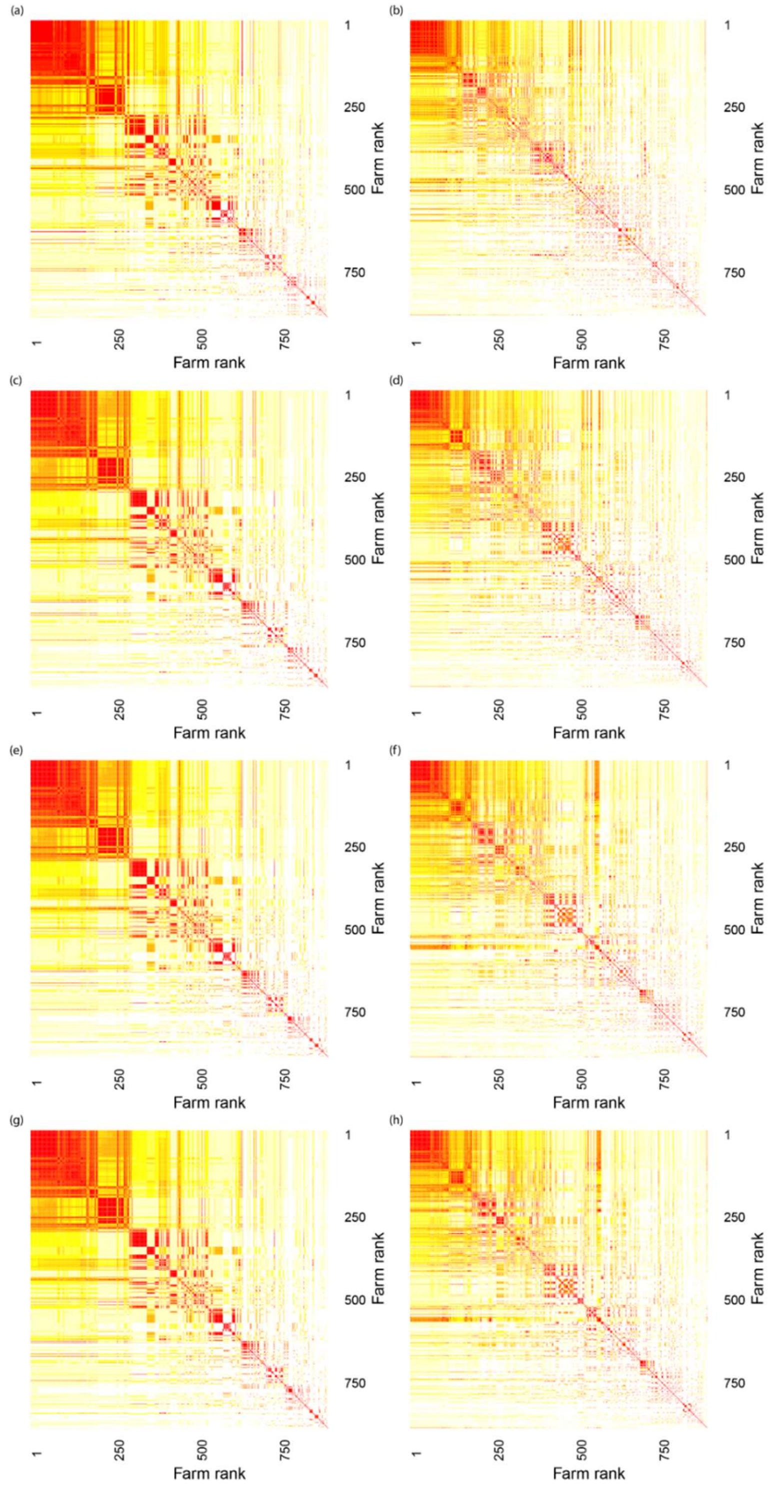

Fig S3.1. Heatmaps showing the overlaps between the invasion paths generated by the seeds in the alternative scenarios. The heatmaps show the effect of the contamination period on the emerge of recurrent invasion paths. Panels (a) and (b): h set to 1 week; panels (c) and (d): h set to 2 weeks; panels (e) and (f) h set to 3 weeks; panels (g) and (h): h set to 4 weeks. In each row, the results obtained with the CCM and TM are shown on the left and on the right, respectively. In each heatmap, the color of the position [i,j] encodes the value of the abundance based version of the Jaccard index computed on the invasion paths generated by the seeds in the positions i and j.

To evaluate the robustness of the clusters of the CCM and TM most influential seeds based on the overlap between their invasion paths in the baseline scenario, we filtered the two initial conditions similarity networks (ICSNs), varying the threshold from 0.8 to 1 (results are shown in Fig S3.2). It emerged that high values of the threshold should be used to detect differences between the set of most influential seeds identified using the CCM. This means that epidemics seeded at these seeds infected sets of nodes almost coincident. Conversely, as regards the TM, even when low threshold values were assumed, the most influential seeds grouped into different clusters, confirming that they generated heterogeneous invasion paths.

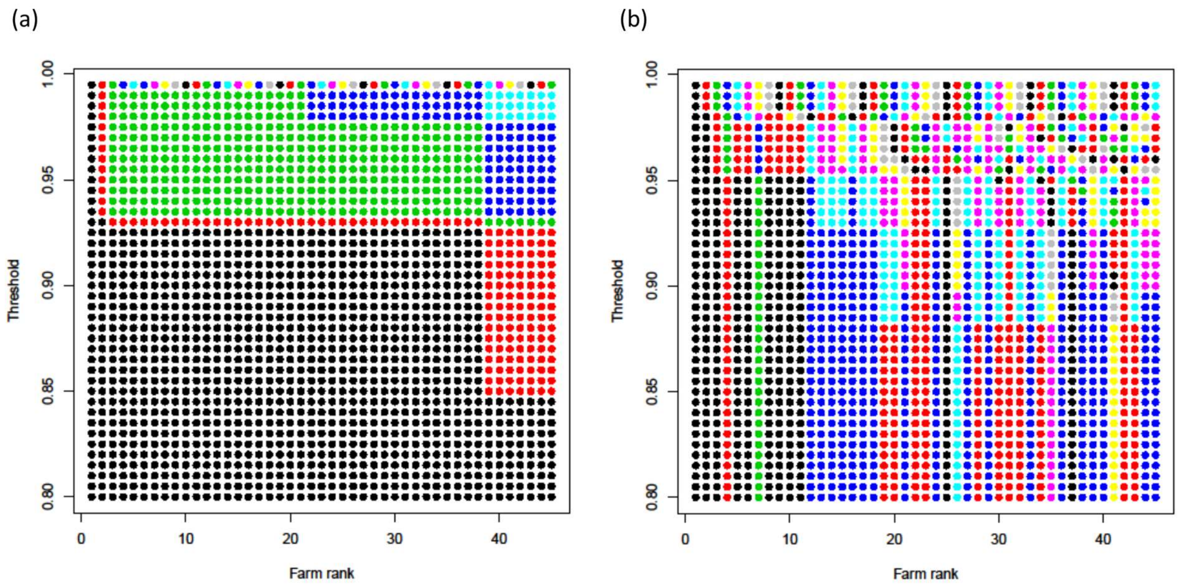

Fig S3.2. CCM and TM seeds' clusters based on the overlap between the invasion paths in the baseline scenario (h = 0). Each panel is a matrix describing how the most influential nodes (sorted on the x-axis according to the median total epidemic size they generated) detected using the CCM (a) and the TM (b) grouped into clusters when the corresponding ICSN was filtered using the threshold values on the y-axis. The color in the position [i, j] encodes the cluster the node j belongs to when the ICSN is filtered using the threshold j. The number of different colors in the i-th row indicates how many different clusters were identified using the corresponding threshold.

As shown in Fig S3.3, when longer contamination periods were assumed, CCM and TM had similar behavior in terms of emergence of recurrent invasion paths. Indeed, in both cases almost all the most influential seeds grouped in the same cluster (except for few isolated nodes), even when high values of the filtering threshold were assumed, e.g., above 0.95.

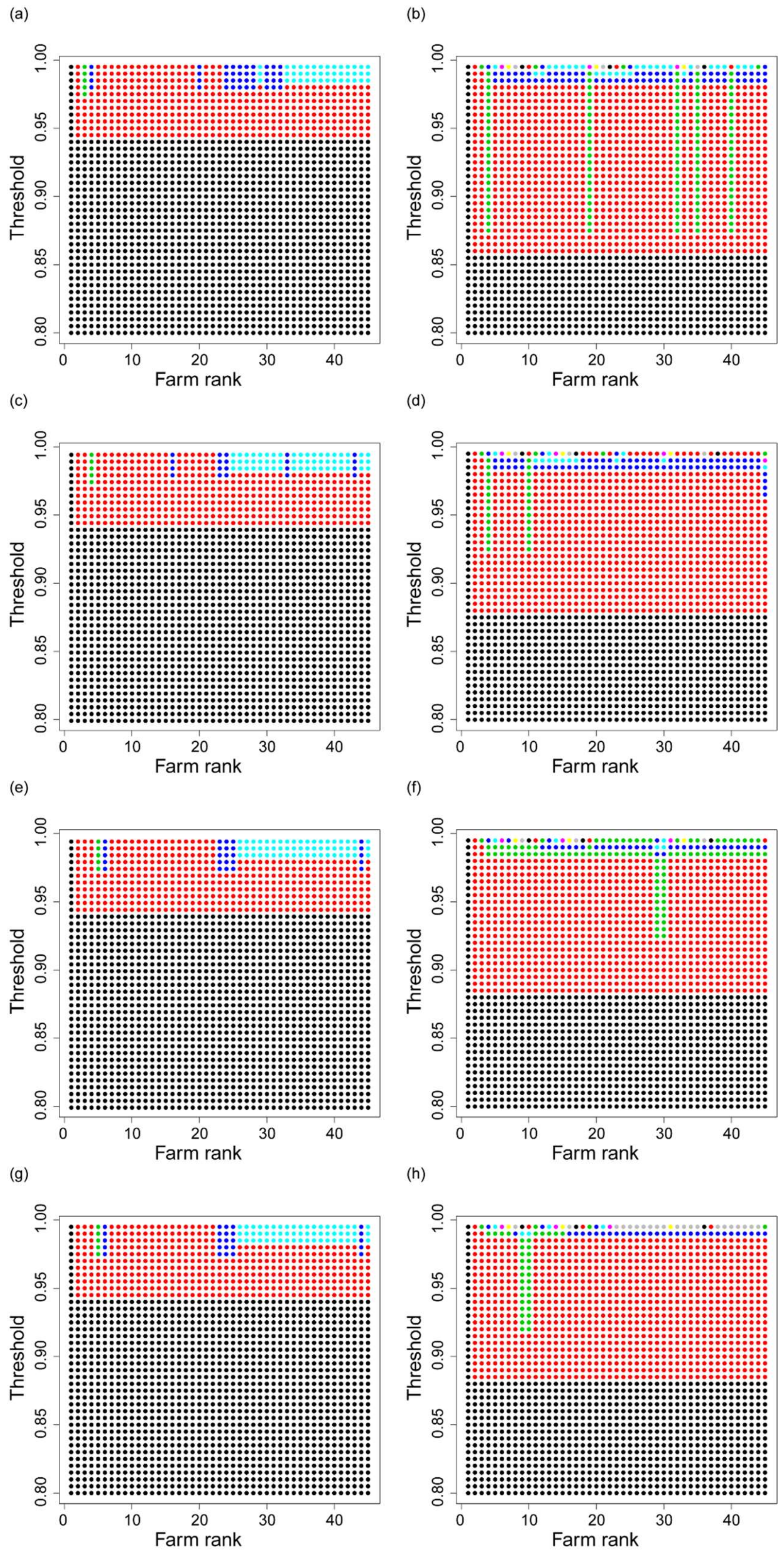

Fig S3.3. Effect of the contamination period on the seeds' clusters based on the overlap between the invasion paths. This figure is analogous to Fig S3.2. Panels (a) and (b): h set to 1 week; panels (c) and (d): h set to 2 weeks; panels (e) and (f) h set to 3 weeks; panels (g) and (h): h set to 4 weeks.
